# Significance of thermal radiation and bioconvection for Williamson nanofluid transportation owing to cone rotation

**DOI:** 10.1038/s41598-022-27118-6

**Published:** 2022-12-31

**Authors:** Sohaib Abdal, Imran Siddique, Sayed M. Eldin, Muhammad Bilal, Sajjad Hussain

**Affiliations:** 1 Department of Mathematics, Khwaja Fareed University of Engineering and Information Technology, Rahim Yar Khan, 64200 Pakistan; 2 School of Mathematics, Northwest University, No. 229 North Taibai Avenue, Xi’an, 7100069 China; 3 Department of Mathematics, University of Management and Technology, Lahore, 54770 Pakistan; 4 Center of Research, Faculty of Engineering, Future University in Egypt, New Cairo, 11835 Egypt; 5 School of Aerospace and Mechanical Engineering, Nanyang Technological University, Singapore, Singapore

**Keywords:** Engineering, Nanoscience and technology, Physics

## Abstract

Numerical investigation for enhancement in thermal distribution of unsteady dynamics of Williamson nanofluids and ordinary nanofluids flow across extending surface of a rotating cone is represented in this communication. Bio-convection of gyrotactic micro-organisms and thermal radiative fluxes with magnetic fields are significant physical aspects of the study. The velocity slip conditions are considered along x and y directions. The leading formulation is transmuted into ordinary differential form via similarity functions. Five coupled equations with non-linear terms are resolved numerically through the utilization of Matlab code for the Runge–Kutta procedure. The parameters of buoyancy ratio and bio-convection Rayleigh number decrease the x-direction velocity. The slip parameter being proportional to viscosity reduces the speed of flow and hence rise in temperature. Also, the temperature rises with the rising values of magnetic field strength, radiative heat transportation, Brownian motion and thermophorsis.

## Introduction

Nanofluids play vital role in the current era because of its enormous diversity and complexity. They are being used in various applications such as in the petroleum industry, medical applications, food processing and many more. Firstly, the concept of nanofluid was given by Choi and Eastman^1^. They discussed the role of thermal conductivity of nano particles in base fluids. Abbas et al.^2^ scrutinized the 2nd-Grade nanofluid flow for unsteady case having thermal radiation and mixed convection. Wang et al.^3^ investigated the effects of nanoparticle accumulation and radiation on the flow of nanofluid. Gowda et al.^4^ computationally studied the effects of Stefan blowing on 2nd grade fluid. Kumar et al.^5^ investigated the influence of activation energy over Darcy-Forchheimer flow of Casson fluid in a porous media. Gowda et al.^6^ studied sedimentation of thermophoretic particles in unsteady hybrid nanofluid. Jyothi et al.^7^ elaborated the effects of thermal radiation on casson fluid for non linear case using Buongiorno’s nanofluid model. Li et al.^8^ analysed thehybrid nanofluid in nonlinear mixed convective flow along with entropy. Yusuf et al.^9^ investigated MHD Williamson nanofluid along with gyrotactic organisms. Prasannakumara^10^ numerically studied transport of heat in Maxwell nanofluid flow. Benos et al.^11^ examined the MHD convection of CNT-Water nanofluid using Hamilton-Crosser model. Sarris et al.^12^ studied the large-eddy simulations (LES) of turbulent and transitional channel flows of a conductive fluid under the effect of a uniform magnetic field. Sarris et al.^13^ presented a study of the flow field and residence times in the anode flow bed of a pilot direct ethanol fuel cell (DEFC) using 3-D numerical flow modelling. Karvelas et al.^14^ studied the micromixing efficiency of particles in heavy metal removal processes. Gowda et al.^15–19^ studied the nanofluid flow for different geometries.

An English mathematician Williamson developed the Williamson fluid model^20^ in 1929 and numerous researchers considered it. Williamson fluid is a non-Newtonian fluid model which has a shear thinning property. Srinivas et al.^21^ explored the importance of lubrication of surfaces and convective boundary conditions in the flow of non-Newtonian Williamson fluid. Abdal et al.^22^ investigated MHD Williamson Maxwell nanofluid over a sheet. Qayyum et al.^23^ studied the Williamson nanofluid flow for radiation and velocity slip. Waqas et al.^24^ studied the Fick’s and Fourier’s concept for heat production in nonlinear convective Williamson nanofluid flow. Chu et al.^25^ studied about the thermal energy of hybrid nanoparticles by engaging chemical reaction and activation energy. Chu et al.^26^ elaborated the properties of thermal radiation, heat generation and the effect of convective boundary conditions. Similar work was done by many researchers^22,27–29^

Bio-convection can be termed as hydrodynamic instability and designs in suspensions of biased swaying microorganisms. Bioconvection has several uses in the field of natural systems and biotechnology. Various researchers uses bio-convection of living microorganisms to explore the behavior of fluid. Ramesh et al.^30^ investigated Maxwell nanofluid having gyrotactic organisms along with nonlinear thermal radiation. Song et al.^31^ discussed the micropolar nanofluid for nonlinear thermal radiation having gyrotactic organisms flow and moreover Applications of modified Darcy law. Farooq et al.^32^ studied the bioconvection in Carreau nanofluid flow having numerous thermal consequences. Song et al.^33^ explored the gyrotactic analysis of Sutterby nanofluid having many thermal features. Chu et al.^34^ Collective effect of Cattaneo-Christov double diffusion and radiative heat flux on gyrotacyic organisms flow of Maxwell liquid. Yahya et al.^29^ scrutinized the thermal characteristics of Williamson Sutterby nanofluid through sponge medium.

This survey of past studies convinced that bioconvection of microorganisms immersed in Williamson nanofluid flow across a rotating cone is rarely discussed. The nanofluid flow across the slip surface of the cone in the presence of magnetic field and thermal radiation adds to the physical aspects of this work. There seems a gap to explore:

(1) The impact of nano particle distribution on flow across a rotating cone.

(2) How does the bioconvection, magnetic field and thermal radiation affect the Williamson nanofluid slip transportation?

The motivation of this work pertains to enhancement of thermal distribution to increase thermal conductivity of base fluid with inclusion of nano-entities. The apprehension of the possible settling of nano-material is dismantled through density gradients of microorganisms. Thus bioconvection is considered along with nanofluid transportation across the cone. These physical aspects with heat and mass flow across cone geometry are practicable in rotational dynamical systems. The results can find applications in the efficient working of heat exchangers, cooling of microelectronics and transfer engines.

## Flow assumption and mathematical formulation

**Figure 1:**
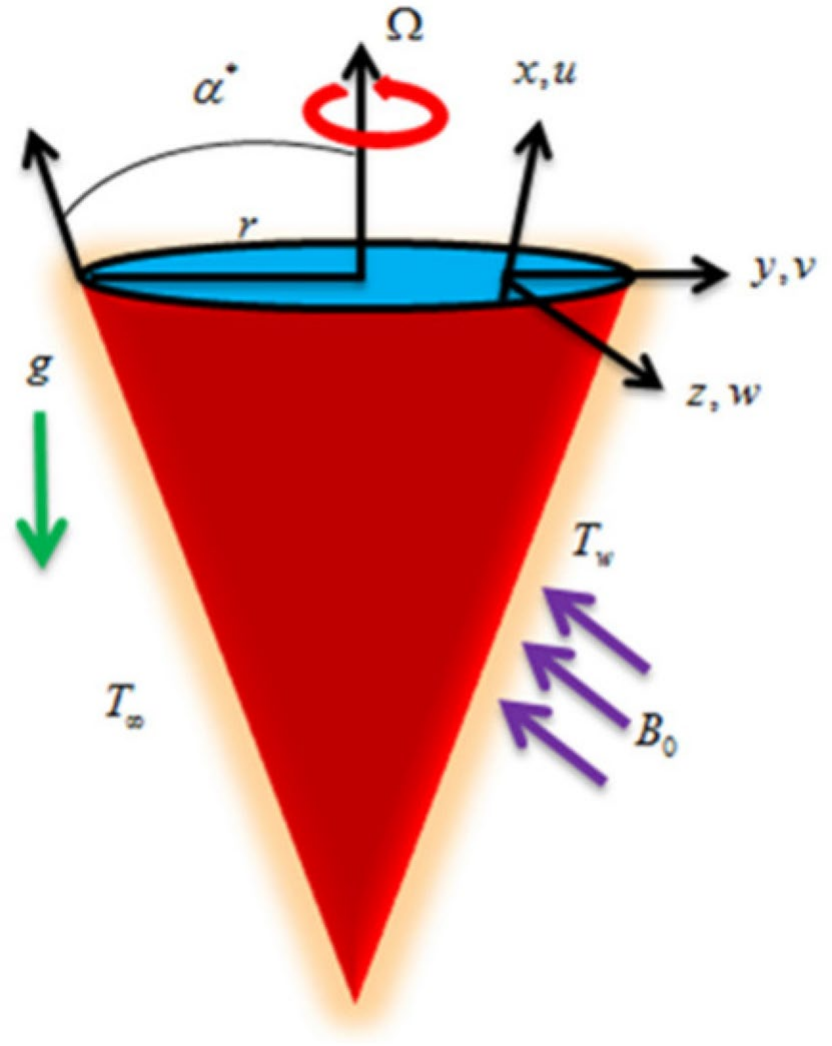
Flow chart.

Considered the unsteady and incompressible Williamson nanofluid with thermal radiation and microorganisms flowing past a rotating cone. Assuming cone rotation velocity as a function of time causes unsteadiness in the flow field. The mass, temperature and microorganisms’ difference in the flow field induce the existence of buoyancy forces. Velocity components *u*, *v* and *w* are along *x*, *y* and *z* directions. Cone rotation is represented by $\Omega$ (see Fig. 1). The flow velocity slips are considered in x and y directions. A magnetic field of strength $B_o$ acts perpendicular to the x-axis. The cone half angle is $\alpha ^*$. The self motile micro-organisms are dilutely mixed with base fluid. The motion of micro-organisms does not depend on the transport of nano particles and vise versa. The temperature, nano particle concentration and micro-organisms have constant wall conditions. Hall effect is taken in to consideration. The formulation of the leading equations is presented as^35–40^.

1 $$\begin{aligned}{} & {} \frac{\partial (xu)}{\partial x}+\frac{\partial (xw)}{\partial z}=0, \end{aligned}$$

2 $$\begin{aligned}{} & {} \frac{\partial u}{\partial t} + u\frac{\partial u}{\partial x}-\frac{v^2}{x}+w\frac{\partial u}{\partial z}= \nu _0 \bigg (\frac{\partial ^2 u}{\partial z^2}+\sqrt{2}\Gamma (\frac{\partial u}{\partial z})\frac{\partial ^2 u}{\partial z^2}\bigg )-\frac{\sigma B_{0}^2(u+mv)}{\rho (1+m^2)}\nonumber \\{} & {} \quad + \frac{1}{\rho }\bigg (\beta _M(1-C_\infty )\rho g (T-T_\infty ) - (\rho _p - \rho ) g (C-C_\infty ) - (n-n_\infty ) g \gamma (\rho _m - \rho )\bigg ), \end{aligned}$$

3 $$\begin{aligned}{} & {} \frac{\partial v}{\partial t} + u\frac{\partial v}{\partial x}+\frac{uv}{x}+w\frac{\partial u}{\partial z}= \nu _0 \bigg (\frac{\partial ^2 v}{\partial z^2}+\sqrt{2}\Gamma (\frac{\partial v}{\partial z})\frac{\partial ^2 v}{\partial z^2}\bigg )-\frac{\sigma B_{0}^2(mu-v)}{\rho (1+m^2)}, \end{aligned}$$

4 $$\begin{aligned}{} & {} \frac{\partial T}{\partial t}+u\frac{\partial T}{\partial x}+w\frac{\partial T}{\partial z}=\frac{k}{\rho c_p}\bigg ((\frac{\partial u}{\partial z})^2+(\frac{\partial v}{\partial z})^2\bigg )+ \frac{16 T^3_\infty \sigma ^*}{3k^* \kappa } \frac{\partial }{\partial z} \frac{\partial T}{\partial z}+\frac{\sigma B_{0}^2(u^2+v^2)}{\rho c_p}+ \tau D_B\frac{\partial C}{\partial z}\frac{\partial T}{\partial z}+\tau \frac{D_T}{T_\infty }(\frac{\partial T}{\partial z})^2, \end{aligned}$$

5 $$\begin{aligned}{} & {} \frac{\partial C}{\partial t}+u\frac{\partial C}{\partial x}+w\frac{\partial C}{\partial z}=D_B\frac{\partial }{\partial z}\frac{\partial C}{\partial z} + \frac{D_T}{T_\infty } \frac{\partial }{\partial z}\frac{\partial T}{\partial z}, \end{aligned}$$

6 $$\begin{aligned}{} & {} \frac{\partial n}{\partial t}+u\frac{\partial n}{\partial x}+ w\frac{\partial n}{\partial z} +\frac{c_b W_c}{C_w-C_\infty }\bigg (\frac{\partial }{\partial z}(n\frac{\partial C}{\partial z})\bigg )=D_m \frac{\partial }{\partial z}\frac{\partial n}{\partial z}. \end{aligned}$$

with suitable boundary conditions

7 $$\begin{aligned} {\left. \begin{aligned} u=N_0 \mu \frac{\partial u}{\partial z}, v=\frac{\Omega x sin\alpha ^*}{1-qt^*}+N_0 \mu \frac{\partial v}{\partial z}, w=0, T=T_w, C=C_w, n=n_w, \ at \ \eta = 0,\\ u = 0, v = 0, T = T_\infty , C = C_\infty , n = n_\infty , \ as \ \eta = \infty . \end{aligned}\right\} } \end{aligned}$$

Using similarity transformations

8 $$\begin{aligned} {\left. \begin{aligned} u = \frac{-\Omega x sin\alpha ^*}{2(1-qt^*)}f'(\eta ), v = \frac{\Omega x sin\alpha ^*}{(1-qt^*)}g(\eta ), w = \sqrt{\frac{\nu _0 \Omega sin\alpha ^*}{(1-qt^*)}}f'(\eta ),\\ \theta (\eta ) = \frac{T-T_\infty }{T_w - T_\infty }, T_w - T_\infty = \frac{x(T_0 - T_\infty )}{L(1-qt^*)^2}, \phi (\eta ) = \frac{C-C_\infty }{C_w - C_\infty }, C_w - C_\infty = \frac{x(C_0 - C_\infty )}{L(1-qt^*)^2},\\ \chi (\eta ) = \frac{n-n_\infty }{n_w - n_\infty }, n_w - n_\infty = \frac{x(n_0 - n_\infty )}{L(1-qt^*)^2}, t^* = (\Omega sin\alpha ^*)t, \eta = \sqrt{\frac{\Omega sin\alpha ^*}{v_0(1-qt^*)}}z. \end{aligned}\right\} } \end{aligned}$$

The transformed ordinary differential equations are:

9 $$\begin{aligned}{} & {} f'''(1-\beta f'')+\frac{f'^2}{2}-2g^2-ff''-S(f'+\frac{\eta }{2}f'')-2\lambda (\theta -Nr\phi -Rb\chi )-\frac{M}{1+m^2}(f'-2mg)= 0, \end{aligned}$$

10 $$\begin{aligned}{} & {} g''(1+\beta g')+gf'-fg'-S(g+\frac{\eta }{2}g')-\frac{M}{1+m^2}(\frac{mf'}{2}+g)= 0, \end{aligned}$$

11 $$\begin{aligned}{} & {} (1+Rd)\theta '' - Pr\bigg (S(2\theta +\frac{\eta }{2}\theta ')-\frac{f'\theta }{2}+f\theta ' \bigg )+Br(\frac{f''^2}{4}+g'^2)+M Br (\frac{f'^2}{4}+g^2) + Nb\theta ' \phi ' + Nt\theta '^2 = 0, \end{aligned}$$

12 $$\begin{aligned}{} & {} \phi '' -Sc \bigg (S(2\phi +\frac{\eta }{2}\phi ')-\frac{f'\phi }{2}+f\phi ' \bigg )+ (\frac{Nt}{Nb})\theta '' = 0, \end{aligned}$$

13 $$\begin{aligned}{} & {} \chi '' - Lb\bigg (S(2\chi +\frac{\eta }{2}\chi ')-\frac{f'\chi }{2}+f\chi ' \bigg ) +Pe[\phi ''(\chi +\delta )+\chi ' \phi '] = 0. \end{aligned}$$

with transformed boundary conditions are:

14 $$\begin{aligned} {\left. \begin{aligned} f(0)=0, \ f'(0) = \Delta _u f''(0),\ g(0)=1+\Delta _u g'(0), \ \theta (0)=1,\ \phi (0)=1,\ \chi (0)=1, \ at \ \eta = 0,\\ f'(\infty )\rightarrow 0,\ g(\infty )\rightarrow 0, \ \theta (\infty )\rightarrow 0, \phi (\infty )\rightarrow 0, \chi (\infty )\rightarrow 0, \ as \ \eta \rightarrow \infty . \end{aligned}\right\} } \end{aligned}$$

Where the non-dimensional parameters are $\beta = \Gamma x \sqrt{\frac{1}{2\nu _0}(\frac{\Omega sin\alpha ^*}{1-qt^*})^3}$ Williamson fluid parameter, $M = \frac{\sigma B_o^2 (1-qt^*)}{\rho \Omega sin\alpha ^*}$ is magnetic parameter, $Nr = \frac{(\rho _p - \rho ) (C-C_\infty )}{\beta _M(1-C_\infty )\rho (T-T_\infty )}$ represents buoyancy ratio parameter, $Rb = \frac{\gamma (\rho _m - \rho ) (n-n_\infty )}{\beta _M(1-C_\infty )\rho (T-T_\infty )}$ is Rayleigh number, $\lambda = \frac{Gr}{Re^2}$ is mixed convection parameter, $Gr = \frac{g \beta _t cos\alpha ^* (T-T_\infty )L^3}{\nu _0^2}$ is Grashof number, $Re = \frac{L^2 \Omega sin\alpha ^*}{\nu _0}$ is Reynolds number, $Sc = \frac{\nu }{D_B}$ represents Schmidt number, $Pr = \frac{k}{\alpha }$ is the Prandtl number, radiation parameter is $Rd = \frac{16 T_\infty ^3 \sigma ^*}{3k*}$, Peclet number is $Pe = \frac{c_b W_c}{\nu _0}$, bioconvection Lewis number is $Lb = \frac{\nu }{D_m}$, Brownian motion parameter is $Nb = \frac{\tau D_B (C-C_\infty )}{(1-qt^*)^2 L}$ and thermophoresis parameter is $Nt = \frac{\tau D_T (T-T_\infty )\Omega sin\alpha ^*}{T_\infty (1-qt^*)^2 L \nu _0}$.

## Physical quantities

### Skin friction coefficient

The coefficient of surface drag is represented by:

$$\begin{aligned} Cf_x = \frac{2\tau _{xz}}{ \rho \left( \frac{\Omega sin\alpha ^*}{1-qt^*}\right) ^2} \end{aligned}$$

where, $\tau _{xz}$ is a shear stress detector and is defined as:

$$\begin{aligned} \tau _{xz} = \mu (1+\beta )\left( 1+\frac{\Gamma }{2}\frac{\partial u}{\partial z}\right) \frac{\partial u}{\partial z} \ \ \ \ at \ \ z = 0 \end{aligned}$$

Applying Eq. (8), the dimensionless formulation of the preceding equation is:

$$\begin{aligned} Cf_x(Re_x)^{ \frac{1}{2}} = -(f''(0)+\beta (f''(0))^2) \end{aligned}$$

also

$$\begin{aligned} Cf_y = \frac{2\tau _{yz}}{ \rho \left( \frac{\Omega sin\alpha ^*}{1-qt^*}\right) ^2} \end{aligned}$$

where, $\tau _{yz}$ is a shear stress detector and is defined as:

$$\begin{aligned} \tau _{yz} = \mu (1+\beta )\left( 1+\frac{\Gamma }{2}\frac{\partial v}{\partial z}\right) \frac{\partial v}{\partial z} \ \ \ \ at \ \ z = 0 \end{aligned}$$

Applying Eq. (8) the dimensionless formulation of the preceding equation is:

$$\begin{aligned} Cf_y(Re_x)^{ \frac{1}{2}} = -(g'(0)+\beta (g'(0))^2) \end{aligned}$$

### Local Nusselt number

The mathematical solution for the heat transfer efficiency relationship is as described in the following:

$$\begin{aligned} Nu_x = \frac{xq_w}{k (T_w - T_\infty )} \end{aligned}$$

The external heat transfer is:

$$\begin{aligned} q_w = - \frac{\partial T}{\partial z} - \frac{16 T_\infty ^3 \sigma ^*}{3k*}\frac{\partial T}{\partial z} \ \ \ \ at \ \ z = 0 \end{aligned}$$

Using Eq. (8), the preceding solution is reduced as follows:

$$\begin{aligned} Nu_x(Re_x)^{-1/2} = -(1-Rd)\theta '(0) \end{aligned}$$

### Sherwood number

It is defined as:

$$\begin{aligned} Sh_x = \frac{xq_m}{k(C_w - C_\infty )} \end{aligned}$$

where $q_m$ stands for surface mass flow and is denoted as::

$$\begin{aligned} q_m = -D_B \frac{\partial C}{\partial z} \ \ \ \ at \ \ z = 0 \end{aligned}$$

Using Eq. (8), the above equation’s non-dimensional version is:

$$\begin{aligned} Sh_x(Re_x)^{-1/2} = -\phi '(0) \end{aligned}$$

### Density of micro-organisms

It is defined as:

15 $$\begin{aligned} Nn_x = \frac{xq_n}{k(n-n_{\infty })} \end{aligned}$$

where $q_n$ identifies the flux of motile microorganisms and is delineated as:

16 $$\begin{aligned} q_n = - D_n(C) \frac{\partial n}{\partial z} \ \ \ \ at \ \ z = 0 \end{aligned}$$

Using Eq. (8), the non-dimensional form of equation is:

$$\begin{aligned} Nn_x(Re_x)^{ \frac{-1}{2}} = - \chi '(0) \end{aligned}$$

## Numerical procedure

This section describes numerical procedure for the leading ordinary differential Eqs. (9)–(13) with boundary conditions (14). Such type of boundary value problems is difficult to solve analytically. Although various numerical approaches are being used for this purpose, yet Range–Kutta (R–K) fourth order method is frequently utilized (see^41–45^). We also hired R-K method for the solution of the problem. To carry out this strategy, the governing Eqs. (9)–(14) are converted into a first-order differential form as shown below:

$$\begin{aligned} y_1'= & {} y_2 \\ y_2'= & {} y_3 \\ y_3'= & {} \frac{-1}{1-\beta f''}\left[ \frac{1}{2} f'^2 - ff'' - 2g^2 - S\left( f'+\frac{1}{2}\eta f''\right) -\frac{M}{1+m^2} (f'-2mg)- 2\lambda (\theta - Nr\phi - Rb\chi )\right] \\ y_4'= & {} y_5 \\ y_5'= & {} \frac{-1}{1-\beta g'}\left[ -fg'+f'g- S\left( g+\frac{1}{2}\eta g'\right) -\frac{M}{1+m^2}\left( g+\frac{mf'}{2}\right) \right] \\ y_6'= & {} y_7 \\ y_7'= & {} \frac{-1}{1+Rd} \left( -Pr\left[ f\theta '-\frac{1}{2}f'\theta +S(2\theta +\frac{1}{2}\eta \theta ')\right] +Pr Ec \left( \frac{f''^2}{4}+g'^2\right) +M Pr Ec \left( \frac{f'^2}{4}6+g^2\right) + Nb\theta ' \phi ' + Nt\theta '^2\right) \\ y_8'= & {} y_9 \\ y_9'= & {} Sc\left[ f\phi '-\frac{1}{2}f'\phi +S\left( 2\phi +\frac{1}{2}\eta \phi '\right) \right] - \left( \frac{Nt}{Nb}\right) \theta '' \\ y_{10}'= & {} y_{11} \\ y_{11}'= & {} Lb\left[ f\chi '-\frac{1}{2}f'\chi +S\left( 2\chi +\frac{1}{2}\eta \chi '\right) \right] -Pe\left[ \phi ''(\chi +\delta )+\chi ' \phi '\right] \end{aligned}$$

along with the boundary conditions:

$$\begin{aligned}{} & {} f(0)= 0, \ f'(0) = \Delta _u f''(0),\ -\Delta _u g'(0)+g(0) = 1, \ \theta (0)=1, \phi (0)=1,\ \chi (0)=1, \ at \ \eta = 0, \\{} & {} f'(\infty )\rightarrow 0,\ g(\infty )\rightarrow 0, \ \theta (\infty )\rightarrow 0, \phi (\infty )\rightarrow 0, \chi (\infty )\rightarrow 0, \ as \ \eta \rightarrow \infty . \end{aligned}$$

This system of first order differential equations is coded in Matlab script.

## Results and discussion

The computations are continued for suitable ranges of the influential parameters; $0.5 \le M \le 2.5$, $0.1 \le \beta \le 2.5$, $0.1 \le m \le 0.5$, $0.1 \le \lambda \le 0.5$, $0.5 \le Nr \le 2.5$, $0.5 \le Rb \le 2.5$, $0.1 \le \Delta _u \le 0.5$, $1.0 \le Br \le 3.0$, $0.1 \le Nb \le 0.5$, $0.01 \le Nt \le 0.05$, $6.0 \le Pr \le 8.0$, $0.1 \le Rd \le 0.5$, $1.0 \le Sc \le 5.0$, $1.0 \le Lb \le 5.0$, $1.0 \le Pe \le 5.0$, $1.0 \le \Omega \le 5.0$. The fixed values for the parameters are chosen arbitrarily $M = 2.0$, $\beta = 0.5$, $m = 1.0$, $\lambda = 0.1$, $Nr = 0.1$, $Rb = 0.1$, $\Delta _u = 1.0$, $Br = 1.0$, $Nb = 0.1$, $Nt = 0.1$, $Pr = 7.0$, $Rd = 0.1$, $Sc = 4.0$, $Lb = 1.0$, $Pe = 1.0$ and $\Omega = 0.3$. Tables 1 and 2 show the comparison of the current numerical study with already published research work (Chamka et al.^35^ and Deebani et al.^36^). There seems a good correlation among the results. Thus numerical approach is validated and the computational procedure is continued.

**Table 1:** The comparative outputs with respect to x.

*Pr*	$\lambda$	Chamka et al.^35^	Deebani et al.^36^	Present Results
0.7	0.0	1.0255	1.022543	1.022535
0.7	1.0	2.2015	2.201024	2.201036
0.7	10.0	8.5041	8.504256	8.504233
10.0	0.0	1.0256	1.025543	1.025561
10.0	1.0	1.5636	1.563001	1.563422
10.0	10.0	2.0201	2.082000	2.024200

**Table 2:** The comparative outputs with respect to y.

*Pr*	$\lambda$	Chamka et al.^35^	Deebani et al.^36^	Present Results
0.7	0.0	0.6158	0.615430	0.615545
0.7	1.0	0.8494	0.849312	0.849462
0.7	10.0	1.3995	1.399221	1.399365
10.0	0.0	0.6158	0.615831	0.615442
10.0	1.0	0.6837	0.683534	0.683664
10.0	10.0	0.9840	0.984555	0.986325

**Figure 2:**
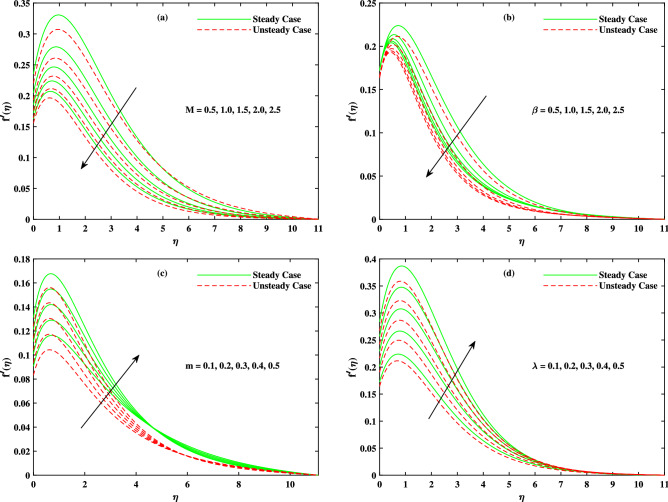
Fluctuation in x-direction velocity $f'(\eta )$ with (**a**) *M*, (**b**) $\beta$, (**c**) *m* and (**d**) $\lambda$.

**Figure 3:**
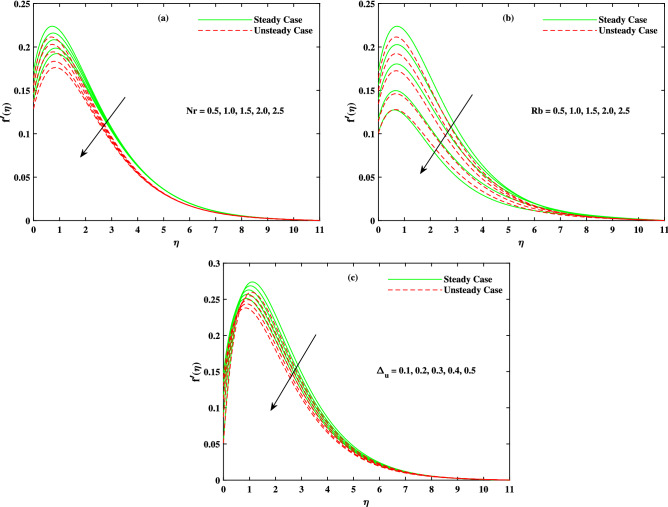
Fluctuation in x-direction velocity $f'(\eta )$ with (**a**) *Nr*, (**b**) *Rb* and (**c**) $\Delta _u$.

**Figure 4:**
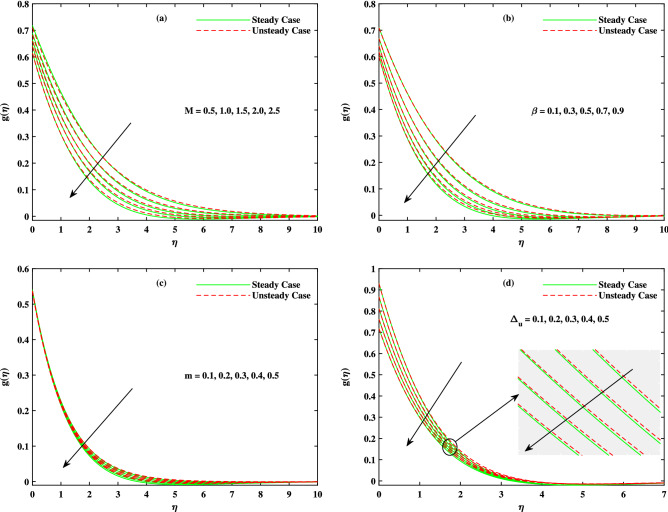
Fluctuation in y-direction velocity $g(\eta )$ with (**a**) *M*, (**b**) $\beta$, (**c**) *m* and (**d**) $\Delta _u$.

**Figure 5:**
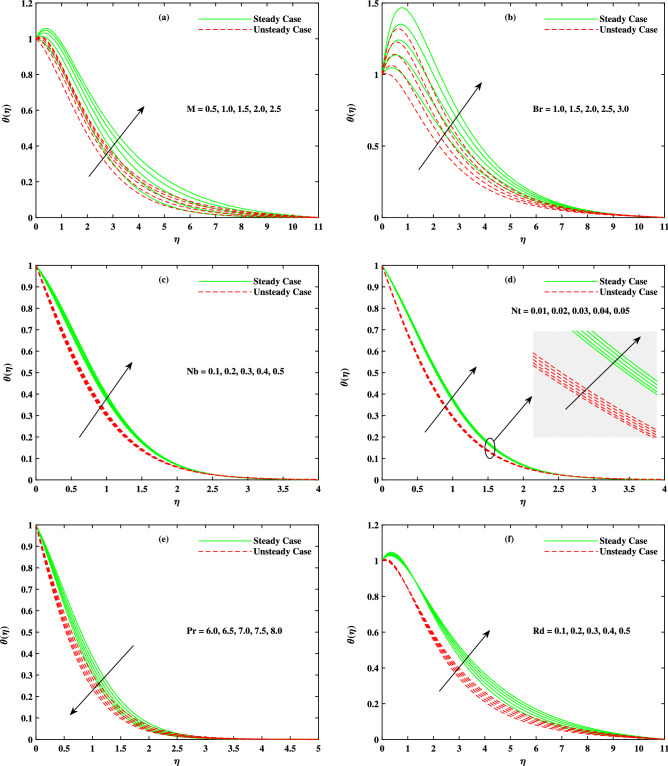
Fluctuation in temperature $\theta (\eta )$ with (**a**) *M*, (**b**) Br, (**c**) *Nb*, (**d**) *Nt*, (**e**) *Pr* and (**f**) *Rd*.

**Figure 6:**
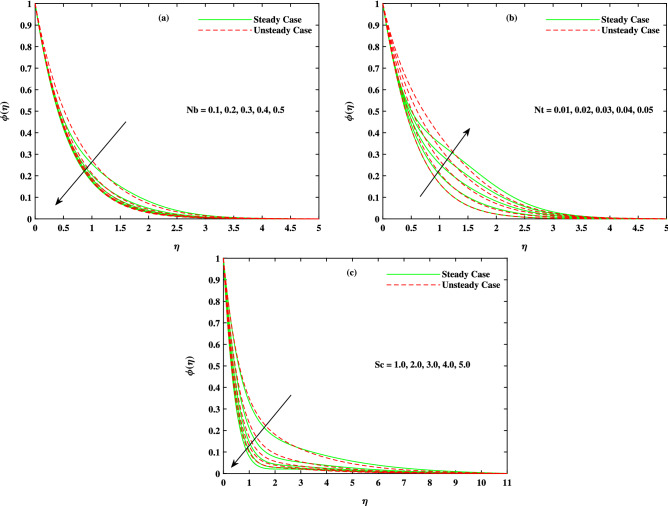
Fluctuation in concentration $\phi (\eta )$ with (**a**) *Nb*, (**b**) *Nt* and (**c**) *Sc*.

**Figure 7:**
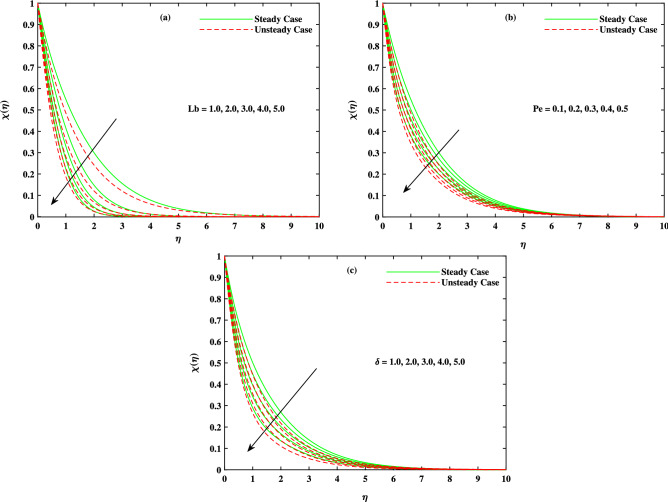
Fluctuation in motile density $\chi (\eta )$ with (**a**) *Lb*, (**b**) *Pe* and (**c**) $\delta$.

It is to mention that throughout the graphs, green solid lines represent the steady case while red dotted lines represent unsteady case. Figure 2a shows the behavior of magnetic parameter *M* on velocity profile. It is seen that velocity decreases when *M* takes larger values. From the figure, it is seen that velocity decreases more rapidly for unsteady case than that of steady case. Physically, the basic reason behind this retardation is the Lorentz force produces resistance to the motion of fluid. Due to this resistance, velocity decreases. Figure 2b shows the effect of $\beta$ on velocity profile. Decreasing behavior is observed in velocity profile when the value of $\beta$ increases. The Williamson fluid parameter $\beta$ is directly related to $\Gamma$, the time relaxation variable and hence retardation of flow is resulted. Opposite behavior for *m* is seen in Fig. 2c. Figure 2d shows the behavior of mixed convection parameter $\lambda$ on velocity profile. It is clearly seen that for both cases steady and unsteady, velocity increases when $\lambda$ increases. This incremented mixed convection causes, the faster flow due to buoyancy forces. The basic phenomenon of this increment in the velocity profile is that when $\lambda$ takes larger values, velocity of the fluid is enhanced. Figure 3a,b,c show the decreasing behavior in velocity profile when buoyancy ratio parameter *Nr*, Rayleigh number *Rb* and $\Delta _u$ increase respectively. The basic phenomenon of this retardation in velocity profile is that there occurs more resistance in horizontal direction of fluid flow with larger values of these parameters. The effect of *M*, $\beta$, *m* and $\Delta _u$ on velocity $g(\eta )$ is observed in Fig. 4. It is clear from the figure that velocity decreases when values of above said non-dimensional parameters increase. Figure 5 shows the impact of *M*, *Br*, *Nb*, *Nt*, *Pr* and *Rd* on temperature profile. It is observed from Fig. 5a,b that temperature increases with the rising values of *M* and *Br*. As mentioned earlier, the fluid velocity decreases against *m*, the kinetic energy is converted in heat energy and hence temperature of fluid is risen. Physically, Brinkman number increase the thermal field of the fluid flow for higher estimations. Due to this, a smaller amount of thermal conduction to the fluid occur. Figure 5c,d show the behavior of *Nb* and *Nt* on temperature profile. From the figure, it is seen that temperature rises with the rising values of *Nb* and *Nt*. The basic concept for increase in temperature due to Brownian motion is that the nanoparticles are directly related with temperature, which means kinetic energy of these particles increases when temperature is enhanced. Also, for thermophoresis parameter, particles move from hotter surface to colder surface, thus temperature of fluid increases. Figure 5e shows the temperature decreases with rising values of *Pr* (Prandtl number). Physically *Pr* is inversely proportional to thermal diffusivity which causes reduction in temperature. Figure 5f shows the effect of radiation parameter *Rd* on temperature profile. It is noted that temperature increases with rising values of *Rd*. The basic reason behind is that a large amount of heat is produced in radiation process. Figure 6 shows the effect of *Nb*, *Nt* and *Sc* on concentration profile. For rising values of *Nt*, the concentration increases rapidly while it goes down for *Nb* and *Sc*. Figure 7 shows the effect of *Lb*, *Pe* and $\delta$ on motile density profile. It is clearly seen that the motile microorganisms profile goes down when the values of *Lb*, *Pe* and $\delta$ are uplifted. The basic reason behind this retardation of *Pe* is that the diffusivity of living microorganisms decreases down when Peclet number takes larger values. The effect of skin friction factor due to different parameters like *M*, $\beta$, *m*, $\lambda$, *Nr*, *Rb* and $\Delta _u$ for both steady and unsteady cases can be seen in Table 3. With the rising values of *M*, skin friction factor increases more for steady case than that of unsteady case values. When $\beta$ value increases, steady case decrease more than unsteady case. As *m* increases, steady case shows more gain in values than unsteady case. When $\lambda$ increases, steady case values increase more than unsteady case values. However, an increase in *Nr* results in decrease in the values of steady case and unsteady case too, but there is more decrease in steady case. For increase in the values of *Rb*, there is an equal amount of decrease in values for both cases. For $\Delta _u$ values, both case values increase equally as $\Delta _u$ increases. Table 4 shows the results of $g'(0)$ for *M*, $\beta$, *m* and $\Delta _u$ for both steady and unsteady cases. It is clearly seen that as *M* increases, the values of steady case increase more than the values of unsteady case. On the other hand, increase in the values of $\beta$, *m* and $\Delta _u$ causes more decrease in the values of steady case than that of unsteady case. Table 5 displays the results for $\theta '(0)$ when *Rd*, *Nb*, *Nt* and *Br* are in action for both cases. It is seen that *Rd* increases more for unsteady case than that of steady case. Contrary the values of *Nb*, *Nt* and *Br* decrease more for unsteady cases than steady cases. In Table 6, the effect of Sherwood number for different parameters *Sc*, *Nb*, *Nt* and $\Gamma$ are shown. As the values of *Sc* and *Nb* increase, Sherwood number increase. As *Nt* value increase, there occur more increase for unsteady case than steady case. For $\Gamma$ values, there is more increase in steady case than unsteady case as $\Gamma$ increases. Table 7 shows *Lb*, *Pe* and $\delta$ results for $\chi '(0)$. As *Lb*, *Pe* and $\delta$ values increase, there is more increase in the values for unsteady cases than that for steady cases.

**Table 3:** Results of skin friction factor –$f''(0)$ in x-direction for various parameters.

*M*	$\beta$	*m*	$\lambda$	*Nr*	*Rb*	$\Delta _u$	$Steady \ Case$	$Unsteady \ Case$
1.0	0.5	1.0	0.1	0.1	0.1	1.0	0.3660	0.3254
1.5							0.4156	0.4007
2.0							0.4724	0.4511
2.0	0.5						0.4724	0.4511
	1.0						0.3178	0.3026
	1.5						0.2648	0.2516
	0.5	0.5					0.3500	0.3308
		1.0					0.4724	0.4511
		1.5					0.5542	0.5297
		1.0	0.1				0.4724	0.4511
			0.2				0.5152	0.4889
			0.3				0.5556	0.5251
			0.1	0.1			0.4724	0.4511
				0.3			0.4636	0.4419
				0.5			0.4549	0.4326
				0.1	0.1		0.4724	0.4724
					0.3		0.4584	0.4584
					0.5		0.4443	0.4443
					0.1	0.1	1.4834	1.4834
						0.5	0.8209	0.8209
						1.0	0.4724	0.4724

**Table 4:** Results of skin friction factor $g'(0)$ in y-direction for various parameters.

*M*	$\beta$	*m*	$\Delta _u$	$Steady \ Case$	$Unsteady \ Case$
1.0	0.5	1.0	1.0	1.1121	1.1260
1.5				1.2176	1.2279
2.0				1.3022	1.3100
2.0	0.5			1.3022	1.3100
	1.0			0.9735	0.9779
	1.5			0.8130	0.8546
	0.5	0.5		1.3612	1.3695
		1.0		1.3022	1.3100
		1.5		1.2349	1.2429
		1.0	0.1	2.0165	2.0468
			0.5	1.6381	1.6536
			1.0	1.3022	1.3100

**Table 5:** Results of Nusselt number –$\theta '(0)$ for various parameters.

*Rd*	*Nb*	*Nt*	*Br*	$Steady \ Case$	$Unsteady \ Case$
0.1	0.1	0.1	1.0	0.5787	1.0386
0.2				0.6186	1.0946
0.3				0.6571	1.1485
0.1	0.1			0.5787	1.0386
	0.2			0.5392	0.9919
	0.3			0.5013	0.9469
	0.1	0.01		0.6031	1.0656
		0.05		0.5922	1.0535
		0.1		0.5787	1.0386
		0.1	0.1	1.0168	1.4133
			0.5	0.8219	1.2468
			1.0	0.5787	1.0386

**Table 6:** Results of Sherwood number –$\phi '(0)$ for various parameters.

*Sc*	*Nb*	*Nt*	$Steady \ Case$	$Unsteady \ Case$
4.0	0.1	0.1	1.6753	1.4135
5.0			1.8858	1.6290
6.0			2.0729	1.8215
4.0	0.1		1.6753	1.4135
	0.2		1.6796	1.5722
	0.3		1.6807	1.6248
	0.1	0.01	1.6568	1.6667
		0.05	1.6620	1.5503
		0.1	1.6753	1.4135

**Table 7:** Results of motile density number –$\chi '(0)$ for various parameters.

*Lb*	*Pe*	$\delta$	$Steady \ Case$	$Unsteady \ Case$
0.1	0.1	0.1	0.3011	0.3188
0.5			0.4357	0.5327
1.0			0.5721	0.7064
1.0	0.1		0.5721	0.7064
	0.2		0.7326	0.8310
	0.3		0.8945	0.9572
	0.1	0.1	0.5721	0.7064
		0.2	0.5862	0.7167
		0.3	0.6002	0.7271

## Conclusions

Numerical application is made for magnetohydrodynamic flow of Williamson nanofluid transport across a rotating cone. Bioconvection of microorganisms and radiative heat transfer mode are incorporated. The salient findings are summarized as below:

- It is observed that velocity $f'(\eta )$ decreases when *M*, $\beta$, *Nr*, *Rb*, and $\Delta _u$ uplifts. Opposite behavior is seen for *m* and $\lambda$.
- it can also be seen that velocity $g'(\eta )$ decreases when *M*, $\beta$, *m* and $\Delta _u$ take larger values.
- When *M*, *Br*, *Nb*, *Nt* and *Rd* takes larger values temperature profile $\theta '(\eta )$ decreases. While $\theta '(\eta )$ increases when *Pr* uplifts.
- It is seen clearly seen that concentration profile $\phi '(\eta )$ decreases when *Nb*, *Sc*, $\Gamma$ take larger values. Concentration profile $\phi '(\eta )$ increases when *Nt* uplift.
- Motile density profile $\chi '(\eta )$ decreases when *Lb*, *Pe* and $\Omega$ take larger values.
- Skin friction factor $f''(0)$ increases when *M*, *m* and $\lambda$ take larger values While decreases when $\beta$, *Nr*, *Rb* and $\Delta _u$ uplifted.
- Skin friction factor $g'(0)$ increases when *M* take larger value while decreases for $\beta$, *m* and $\Delta _u$.
- Nusselt number $\theta '(0)$ decreases when *Nb*, *Nt* and *Br* take larger values but rises when *Rd* upsurge.
- Sherwood number $\phi '(0)$ increases when *Sc*, *Nb* and *Nt* uplifted.
- Motile density number $\chi '(0)$ increases when *Lb*, *Pe* and $\delta$ take larger values.

## Future work

This work can be further studied for hybrid nanofluid flow across stretching and rotating cone.

## Data Availability

The datasets used during the current study available from the corresponding author on reasonable request.

## Latin symbols

*u*, *v*, *w*: Velocity components (m/s)
x, y, z: Cartesian coordinates (m)
$B_o$: Magnetic field strength (T)
C: Concentration of nanoparticles
T: Temperature of nanoparticles (K)
n: Micro-organisms distribution
g: Gravity (m/s$$^2$$)
$k^*$: Mean absorption co-efficient (1/m)
k: Thermal conductivity (W/m K)
$\rho _pC_p$: Effective heat capacity (J/K)
$D_B$: Brownian diffusion coefficient (m$^2/$s)
$D_T$: Thermophoresis diffusion coefficient (m$^2/$s)
$D_m$: Diffusivity of microrganisms (m$^2/$s)
$$T_{\infty }$$: Ambient temperature (K)
$C_{\infty }$: Ambient concentration of nanoparticles
$n_{\infty }$: Ambient micro-organisms distribution
$$c_b$$: Chemotaxis constant (m)
$W_c$: Speed of gyrotactic cell (m/s)
$q_w$: Heat tranfer rate (W/m$^2$)
M: Magnetic parameter
Nr: Buoyancy ratio parameter
Rb: Rayleigh number
Rd: Radiation parameter
Pr: Prandtl number
Nb: Brownian motion parameter
Nt: Thermophoresis parameter
Lb: Bio-convection Lewis number
Pe: Peclet number

## Greek symbols

$\alpha ^*$: Semi vertical angle
$\beta$: Williamson fluid parameter
$$\beta _c$$: Concentration coefficient
$$\beta _t$$: Thermal coefficient
$$\nu _0$$: Kinematic viscosity (m$$^2/$$s)
$$\sigma$$: Electrical conductivity (S/m)
$\sigma ^*$: Stefan–Boltzmann constant (W m$^{-2}$ K$^{-4}$)
$$\rho$$: Density (kg/m$$^3$$)
$$\eta$$: Similarity variable
$$\theta$$: Similarity temperature
$\phi$: Similarity concentration of nanoparticles
$\chi$: Similarity density of micro-organisms
$\delta$: Microorganisms concentration difference
